# Incisional hernia repair with plication and utilization of Botox injections: First case report from Saudi Arabia for a 19‐year‐old female

**DOI:** 10.1002/ccr3.1984

**Published:** 2019-01-08

**Authors:** Talal Hijji, Abdullah AlShammari, Alanoud AlHammad, Ghadah AlKhalefah, Fuad Hashem, Salha Almomen, Mohammad Aburahmah

**Affiliations:** ^1^ College of Medicine Alfaisal University Riyadh Saudi Arabia; ^2^ King Faisal Specialist Hospital and Research Center (KFSH&RC) Riyadh Saudi Arabia

**Keywords:** abdominal wall defects, Botox injection, surgical hernia repair, ventral hernia

## Abstract

This article reports on the use of Botox preoperatively for the treatment of a complex ventral hernia which would have typically been treated with component separation technique. The case demonstrates that using the recently developed technique can aid in performing a tension‐free hernia repair with potentially lower complication and recurrence rates.

## 1 INTRODUCTION

Ventral hernia is one of the most common and challenging abdominal wall defects. We are reporting the first case utilizing BTA as an adjunct for surgical hernia repair in Saudi Arabia. Although promising, safety, efficacy, and feasibility, BTA would need to be further evaluated before its use becomes wide spread.

Ventral/incisional hernia is one of the most common and challenging abdominal wall defects faced by surgeons.^1^ Surgical techniques like component separation (CST) were developed to treat difficult ventral hernias, but those are not without complications; wound infection and lateral abdominal wall weakness are some of the associated surgical morbidities.^2^

Complex hernias may be challenging and need experienced hernia surgeons who are familiar with dealing with multiple forms of abdominal defects especially cases with multiple comorbidities, laparotomies, previous repairs or loss of domain that would need restoring the integrity of the abdominal wall. This is because after laparotomy, there is a disturbance of the dynamic forces of the abdominal wall, most notably lateral traction and migration of midline structures which enhances the hernia defect.^3^

Surgical repair of abdominal wall hernias alongside the injection of Botulinum toxin A (BTA) to lateral abdominal musculature is a new therapeutic concept that may preclude the need for CST for the repair of large abdominal wall defects.^4^ The goal was to perform a tension‐free closure, with abdominal wall dynamic stability while optimizing aesthetic appearance. We are reporting the first case utilizing BTA as an adjunct for surgical hernia repair in Saudi Arabia performed in January 2017.

## 2 CASE PRESENTATION

Our patient is a 19‐year‐old girl, with a long surgical history of multiple complicated abdominal surgeries. She was diagnosed with Primary Hyperoxalosis for which she underwent same setting liver and kidney transplant. A few years afterward, she was diagnosed with intestinal type diffuse large B‐cell lymphoma. After that, she had an ileocecal mass that obstructs the bowel lumen and required oncological resection with right hemicolectomy and anastomosis which was further complicated by anastomotic leakage, wound dehiscence and intra‐abdominal sepsis thus requiring emergency exploratory laparotomy and Hartmann's procedure with end ileostomy. The patient then underwent laparotomy, Hartmann's revision and ileostomy site closure. For the purpose of graft preservation, the patient was on long‐term immunosuppressants.

After more than a year from the last surgery, she developed a central abdominal bulge at the site of her laparotomy scar; she had midline incisional hernia. Due to the long history of laparotomies and multiple surgical procedures, a multidisciplinary approach was necessary, and a surgical treatment plan was set.

### 2.1 Investigations

**Figure 1:**
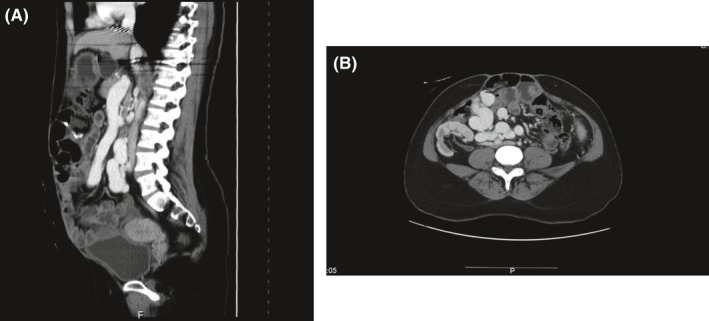
A, B, Computed tomography showing midline fascial defect

An enhanced CT scan was performed and showed thinning of the anterior abdominal wall muscles with severe atrophy of the left rectus abdominis muscle with rectus diastasis of around 10 cm distance (Figure 1).

### 2.2 Treatment

#### 2.2.1 Botox injection

**Figure 2:**
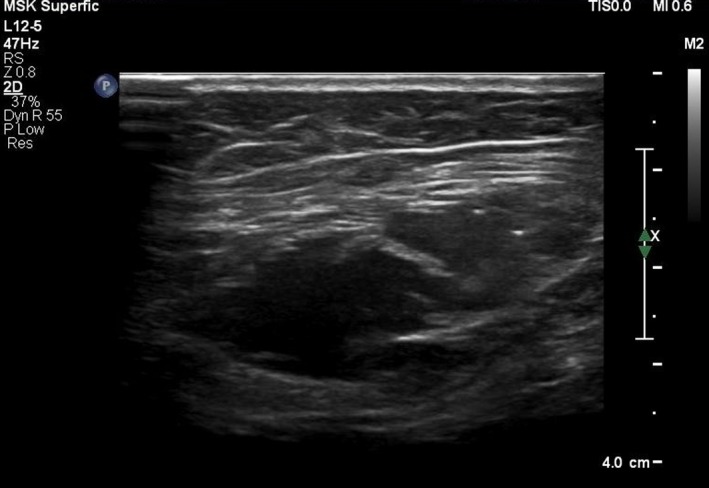
Ultrasound guided injection of 300 U of Botox along the superficial fascia of the transversus abdominis, internal and external oblique muscles bilaterally at three levels; above the iliac crest, at the level of the umbilicus, and below the costal margin

The injection was performed by the interventional radiologist with the patient in supine position under ultrasound guidance (Figure 2). Injection sites were marked at the anterior axillary line between the costal margin and anterior superior iliac spine according to the technique described by Smoot et al.^5^ The area was prepped and draped in a sterile technique which was followed by application of local anesthesia in the form of 1% lidocaine at the skin of injection sites. Under ultrasound guidance, BTA was injected at the three sites on either side of the abdomen. The patient received a total of 300 units of BTA diluted in 150 mL of 0.9% saline with a concentration of 2 units/mL. Each of the six injection sites received a volume of 25 mL. Each of the three injection sites on either side of abdomen were used to target the external oblique, internal oblique, and transversus abdominis muscles. After the procedure, the patient recovered smoothly and was discharged home the next day to return for surgery after 3 weeks.

#### 2.2.2 Injection sites

1. Above the iliac crest, transversus abdominis at anterior axillary line.
2. Mid abdomen, internal oblique at mid axillary line.
3. Below costal margin, external oblique at anterior axillary line.

#### 2.2.3 Surgery

**Figure 3:**
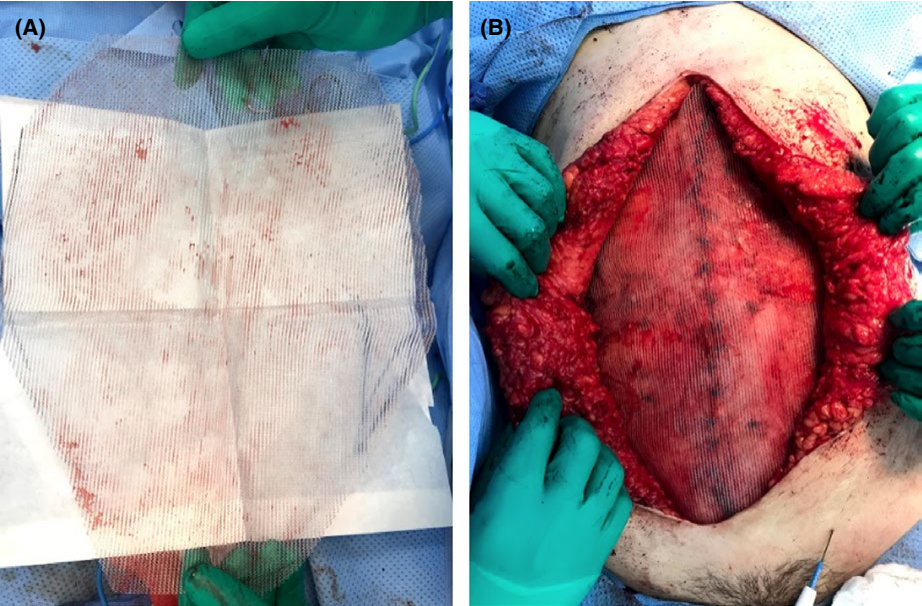
Mesh cut to size and fixed in place using interrupter sutures

Patient went for surgery 3 weeks after Botox injections. The procedure started by infiltrating normal saline for subcutaneous hydro‐dissection followed by removing the old scar at the midline. De‐epithelization was continued just beneath the skin to raise it above the adherent bowel underneath until reaching the normal fascia on both sides of the abdomen. Then, the abdominal flap was raised in the subscarpal plane above the fascia. Closure of the defect and plication of the recti were done followed by placement of sized on‐lay fully resorbable monofilament mesh (Figure 3).

**Figure 4:**
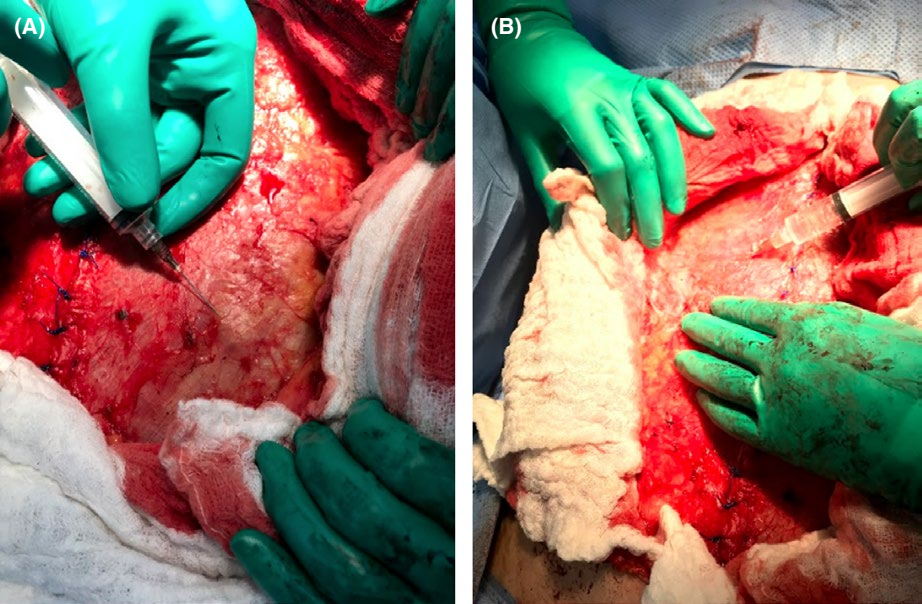
A, B, 300 U of Botox injected on both sides at the anterior axillary line on different levels

**Figure 5:**
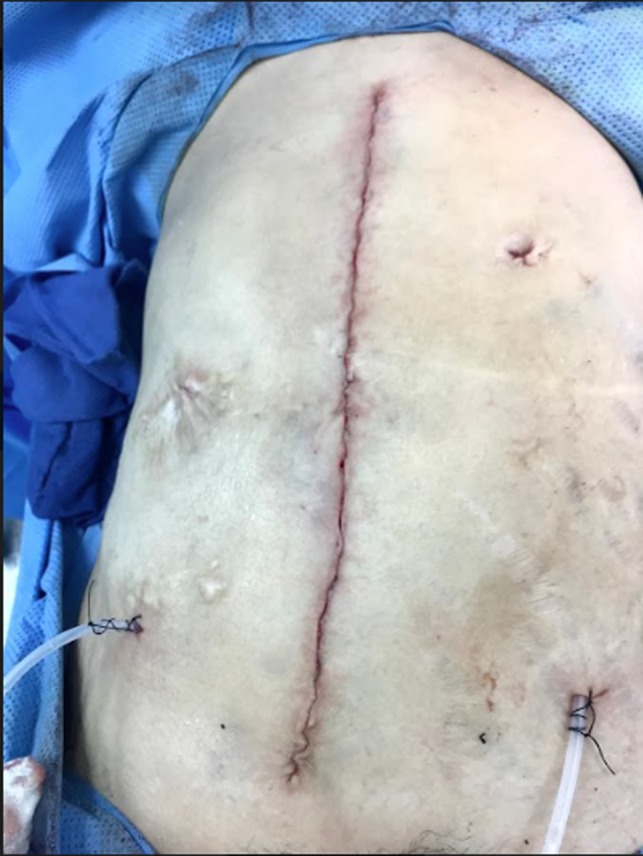
Skin closure

To further relax the lateral abdominal muscles, a total of 200 units of BTA diluted in 8 mL of normal saline were infiltrated at the same previously injected sites (Figure 4). Drains were placed bilaterally above the mesh. Subcutaneous tissue was approximated with interrupted sutures in two layers. Skin was closed with 4‐0 Monocryl in subcuticular fashion (Figure 5). Patient had no complications and was followed up for the next 18 months with no recurrence.

## 3 DISCUSSION

The component separation technique (CST) was first developed by Ramirez in 1991, to facilitate primary closure of large midline abdominal wall defects, such as ventral hernias or resection defects.^2^ Component separation is based on medialization of the rectus abdominis complex without denervating or devascularizing of the musculature by bilateral division of the external oblique aponeurosis at a point lateral to the linea semilunaris.^1^, ^2^ It has been stated that the medial advancement of the epigastrium was up to 5 cm per side, 10 cm at the waistline, and 3 cm in the suprapubic area.^2^ While effective, this technique necessitates large subcutaneous flaps and is associated with significant wound complications including infection, intra‐abdominal abscess, enter cutaneous fistula, and ventral hernia formation.^6^, ^7^ Although these complications could be reduced by providing tension in the midline with negative pressure dressing to offset the traction created by the lateral abdominal wall,^8^ ventral hernia formation rates were still substantial due to the invasiveness of the technique.^9^, ^10^ The use of minimally invasive CST presented a promising option for decreasing complications^11^; however, it was also associated with high recurrence rates.^12^

Botulinum toxin A (BTA) is a neurotoxic protein produced by Clostridium botulinum, which causes reversible (Temporary) muscular paralysis by inhibiting the release of acetylcholine at the neuromuscular junction.^4^ It is given intramuscularly with maximum effect seen within 2 weeks of administration and lasts for about 2.5 months.^13^ Presently, it is used in a wide range of clinical conditions such as skin wrinkles, spastic muscle disorders, hyperhidrosis, and bladder dysfunction and has various other cosmetic^14^ and non‐cosmetic^15^ uses. It's now evident that its flaccid paralysis inducing properties could provide a temporary decreased midline abdominal wall tension. This allows for a pharmacologically safe, reversible alternative for CST while avoiding permanent division to aponeurotic tissue to perform primary fascial closure.^16^

The potential use of BTA as an adjunct for abdominal wall closure was first theorized in 2006 when a study on rats successfully increased intra‐abdominal volume and decreased pressure by paralyzing abdominal wall muscles, thus diminishing transverse hernia defect.^17^ The attempted use of novel technique involving the application of BTA before abdominal wall hernia reconstruction was first reported in 2009, in a study involving 12 patients.^18^ The technique proved useful as the paralysis of lateral muscles and hernia defect reduction (mean of 5.25 ± 2.32 cm) allowed a lower tension closure 4 weeks after BTA application. No recurrence was noted at 9‐month follow‐up. Another prospective study done in Australia studied the effect of preoperative BTA injections on eight patients before attempting surgical repair of recurrent abdominal wall hernias, by injecting 50 unites of BTA at three sites bilaterally (Total of 300 U) to lateral abdominal muscles 2 weeks pre op. Results showed all patients had successful reduction of hernias with no complications and with no early recurrence.^19^ Furthermore, the use of BTA was found to significantly decrease the need for opioid analgesia postoperatively compared to controls, with no difference in complication rates, hospital stay, and recurrence rate in a later study.^20^

Several studies have subsequently been done in order to assess efficacy, complications, and ideal dosage and technique, but none are randomized control trials. The cohorts are investigated in a systemic review involving 133 patients receiving BTA as an adjunct to surgical repair of abdominal incisional hernias.^4^ The studies varied in BTA doses from 300 to 500 U, and injection sites of 3 and 5 per laterality. In total, 83.5% of the patients achieved primary fascial closure, CST was still necessary in 24.1% of patients. Only two patients developed hernia recurrence, no complications were attributed to the use of BTA. However, current evidence was not sufficient to determine optimal dosing, timing, and patient selection as well as to predict when CST would need to be used. The authors conclude that although promising, safety, efficacy, and feasibility, BTA would need to be further evaluated before its use becomes wide spread.

## CONFLICT OF INTEREST

None declared.

## AUTHOR CONTRIBUTION

TH: involved in data collection and manuscript writing. AA: involved in data collection, manuscript writing, and review. AA and GA: reviewed the literature. FH: involved in study design, manuscript review, and case performance. SA: wrote and reviewed the manuscript. MA: involved in study design, manuscript review, and case performance.
